# Supplementary material for: Three-Dimensional Printable Photocurable Elastomer Composed of Hydroxyethyl Acrylate and Hydroxy Fatty Acid Derived from Waste Cooking Oil: An Innovative Strategy for Sustainable, Highly Flexible Resin Development
Source: Molecules. 2025 Oct 6;30(19):4000. doi: 10.3390/molecules30194000 (PMC12525962; doi:10.3390/molecules30194000)
Supplement: Supplementary file 1 [file molecules-30-04000-s001.zip › molecules-3833349-supplementary.pdf]

Supplementary Materials

3D printable photocurable elastomer composed of hydroxyethyl acrylate and hydroxy fatty acid derived from waste cooking oil: An innovative strategy for sustainable, highly flexible resin development

Fangping shen, Chuanyang Tang, Yang Yang, Guangzhi Qin, Minghui Li, Haitian Jiang,

*Mengyao Wu and Shuoping Chen**

College of Materials Science and Engineering, Guilin University of Technology, Guilin 541004, China; fangpingshen_221@163.com (F.S.); 2120230405@glut.edu.cn (C.T.); 1020230205@glut.edu.cn (Y.Y.); 1020230210@glut.edu.cn (G.Q.); 2120230354 @glut.edu.cn (M.L.); 1020240199@glut.edu.cn (H.J.); 2120240427@glut.edu.cn (M.W.);

* Correspondence: chenshuoping_777@163.com (S.C.); Tel.: +86-773-5896672; Fax: +86-773-5896671

Number of pages: 28

Number of tables: 10

Number of figures: 8

Number of Video:3

List

Section S1. Cost analysis for WHFA/HEA resin (A2 Sample).

Section S2. Characterization.

Table S1. The thermogravimetric datas of WHFA/HEA resins and pure HEA.

Table S2. The glass transition temperatures (T_g) of WHFA/HEA resins and pure HEA.

Table S3. Tensile data of WHFA/HEA resins with varying WHFA content.

Table S4. Tensile data of of A2 elastomer in both pre-welding and post-welding states.

Table S5. Tensile data of A2 elastomer across two reprocessing cycles.

Table S6. Interfacial adhesion toughness data of pristine HEA and A2 elastomer on diverse substrate materials.

Table S7. Biodegradation data of A2 elastomer and pristine HEA during soil burial.

Table S8. Fatty acid compositions (wt.%) of WCO. Three main types of fatty acids: saturated (C n:0), monounsaturated (C n:1), and polyunsaturated with two or three double bonds (C n:2/3).

Table S9. The Lovibond color codes of WCO, E-WCO, WHFA and Liquid WHFA/HEA resin.

Table S10. The viscosities of liquid WHFA/HEA resins and other control samples at room temperature.

Figure S1. Full XPS spectra WHFA/HEA resin (A2 sample) and pure HEA.

Figure S2. DTG curves of WHFA-HEA elastomers and pure HEA.

Figure S3. DSC curves of WHFA/HEA elastomer and pure HEA: (a) A1 sample, (b) A2 sample, (c) A3 sample, (d) A4 sample and (e) pure HEA.

Figure S4. lnE-C_d plots of WHFA/HEA elastomer and other control samples: (a) A1 sample, (b) A2 sample, (c) A3 sample, (d) A4 sample, (e) E-WCO/HEA resin and (f) pure HEA.

Figure S5. Stress-strain curves: (a) WHFA/HEA elastomers with different HEA contents (A1-A4 samples); pure HEA and E-WCO/HEA were used as control samples; (b) A2 elastomer before and after 12 hours of welding; (c) A2 elastomer before and after different physical reprocessing cycles.

Figure S6. DMA curves of (a) WHFA/HEA elastomer (A2 sample) and pure HEA (b).

Figure S7. Photograph of WCO, E-WCO, WHFA (liquid form, melted at 60 °C) and liquid WHFA/HEA resin showing the difference in their colors.

Figure S8. CAD model diagram of 1BA dumbbell-shaped spline according to the China Standard GB/T 1040.2-2006.

Video S1. Video presentation of the deformation recovery characteristics of a 3D printed sword made from A2 elastomer at room temperature.

Video S2. Video presentation of the cyclic tensile behavior of a 3D printed A2 elastomer specimens under variable stress-strain regimes (10× accelerated playback).

Video S3. Video presentation of the reversible adhesion performance of a cubic structure under cyclic loading-unloading conditions. The cubic structure was printed using commercial resin, but the adhesive surface used a coated cured WHFA/HEA elastomer (A2 sample) as a pressure-sensitive adhesive layer.

Section S1. Cost analysis for WHFA/HEA resin (A2 Sample).

1. Composition and total mass of the A2 sample

Based on the synthesis formulation provided in Table 3, the A2 sample consists of the following components:WHFA: 15 g; HEA: 45 g; Irgacure 819: 1.8 g; DMAB: 1.8 g

The total mass of the mixture is calculated as: 15+45+1.8+1.8= 63.6 g

2. Unit prices of raw materials (USD/kg)

The following market prices were used for cost estimation:

HEA (2-Hydroxyethyl acrylate): $8.0/kg (industrial grade, bulk price)

Irgacure 819: $150.0/kg (photoinitiator, high purity)

DMAB (4-Dimethylaminobenzaldehyde): $100.0/kg (chemical promoter)

WHFA (WCO-based hydroxy fatty acid): WHFA is synthesized from waste cooking oil (WCO), which is low-cost (~$0.3/kg or often free). Considering reagent consumption (H_2O_2, acetic acid, sulfuric acid, urea, NaOH, HCl) and energy input, the production cost of WHFA is estimated at $1.5/kg at lab scale. Scaling-up could further reduce this cost.

3. Material cost for 63.6 g of A2 resin

Cost of WHFA = (15/1000) kg × $1.5/kg = $0.0225

Cost of HEA = (45/1000) kg × $8.0/kg = $0.36

Cost of Irgacure 819 = (1.8/1000) kg × $150.0/kg = $0.27

Cost of DMAB = (1.8/1000) kg × $100.0/kg = $0.18

Total material cost for 63.6 g = $0.0225 + $0.36 + $0.27 + $0.18 = $0.8325

4. Cost per Kilogram of A2 resin

The cost per kilogram is calculated as follows: $0.8325 / (63.6 / 1000) kg = $13.09/kg

5. Comparison and implications

Commercial petroleum-based flexible photocurable resins typically range from $50–100/kg. The estimated raw material cost of the WHFA/HEA resin ($13.09/kg) demonstrates significant economic advantage and potential for sustainable application in VAT photopolymerization.

This cost evaluation considers only direct material expenses. Further reductions could be achieved through scaled-up production, optimized synthesis, and recovery of reagents.

Thus, the A2 formulation (WHFA: HEA = 1:3) exhibits a competitive material cost structure, highlighting the economic viability of using waste cooking oil-derived components in high-value 3D printing applications.

Section S2. Characterization.

The physicochemical and functional properties of the synthesized WHFA/HEA photocurable elastomer were systematically characterized using established analytical protocols outlined in our previous work[1, 2].

(1) Chemical and physical characterization

The iodine value of WCO was quantified following GB/T 5532-2008. Fatty acid composition analysis was performed through gas chromatography-mass spectrometry (GC-MS, Agilent 7890-5979, Santa Clara, USA) after methyl ester derivatization in accordance with GB 5009.168-2016. Optical properties of WCO, E-WCO and WHFA were evaluated using a LABO-HUB WSL-2 Lovibond tintometer (Xinrui, Shanghai, China).

(2) Spectroscopic analysis

FT-IR spectra (400–4000 cm^{-1}, 4 cm^{-1} resolution) were recorded on a Nicolet 6700 spectrometer (Thermo Fisher Scientific, Waltham, USA) using KBr pelletized samples. Double bond conversion (DC) during photocuring was calculated via[3]:

$$DC=(1-\frac{(A_a^{C=C}/A_a^{C=O})}{(A_b^{C=C}/A_b^{C=O})})\times 100\% \quad (1)$$

Where the pre-irradiation (A_b) and post-irradiation (A_a) absorbance ratios were derived from in-plane bending vibrations of −CH= group (814 cm^{-1}) and C=O stretching (1724 cm^{-1}) peaks. The X-ray photoelectron spectroscopy (XPS) of the cured resin was carried out with an ESCALAB 250Xi X-ray photoelectron spectrometer (Thermofisher, Waltham, USA) with an Al Kα X-ray as the stimulating source.

(3) Migration rate

The migration test of WHFA as a plasticizer was performed according to the following procedure: the WHFA/HEA photocurable elastomers, containing different concentrations of WHFA, were exposed to an isothermal temperature of 135 °C in an oven (Yuejin Medical Device, Shanghai, China) and their weights were measured at predetermined intervals. The migration rate of the plasticizer, expressed as plasticizer weight loss (ws, %), was quantitatively calculated using Equation 2[4]:

$$w_s=\frac{m_0-m_t}{m_0}\times 100\% \quad (2)$$

Additionally, the migration rate of sample A2 at 100°C, 60°C, and 25°C was determined in a similar manner, with only the oven temperature being adjusted.

(4) Thermal behavior

The thermogravimetric analysis was carried out with a TG 209 F1 Libra thermal gravimetric analyzer (Netzsch, Selb, Germany) at a heating rate of 10 °C/min in the range of 30−650 °C under a nitrogen gas atmosphere. The tests of differential scanning calorimetry (DSC) were performed on a DSC 204/2920 differential scanning calorimeter (TA Instruments, New Castle, USA) equipped with a refrigerated cooling system. After equilibrating the samples at −80 °C for 1 min, The temperature was raised from −80 ℃ to 140 ℃ at a rate of 10 ℃ per minute, and then the heating trajectory was recorded to test the glass transition temperature (T_g) of resin.

(5) 3D printing performance

The 3D printing performance of WCO-derived photocurable pressure-sensitive adhesives (elastomer s) was evaluated through determination of penetration depth (D_p) and critical exposure energy (E_c). The absorption of irradiation light by liquid photoresins generally conforms to the Beer-Lambert law, whereby light energy exhibits negative exponential attenuation along the irradiation depth. When the UV exposure exceeds a specific threshold (E_c), the photoresin undergoes phase transition from liquid to solid state. The curing depth (C_d) can be mathematically described by the following equation[5]:

$$C_d=D_p\times \ln\frac{E}{E_c} \quad (3)$$

$$C_d=D_p \ln E\text{-}D_p \ln E_c \quad (4)$$

Where C_d represents curing depth (mm), D_p denotes penetration depth (mm), E_c signifies critical exposure energy (mJ/cm²), and E indicates incident exposure energy (mJ/cm²).

Experimental measurements were conducted using a Photon Mono 2 LCD 3D printer (Anycubic, Shenzhen, China) to cure WCO-derived photocurable elastomer s and control samples under varying exposure durations. This process generated cured films with different thicknesses. Subsequent determination of curing depths and corresponding incident exposure energies enabled the construction of lnE-C_d plots according to Equation (4). Nonlinear fitting through Origin software yielded D_p and E_c values for each sample.

The printing accuracy was assessed by examining the microstructure of 3D-printed specimens using a Leica MC170 HD metallurgical microscope (Leica Microsystems, Germany). This microscopic analysis provided quantitative evaluation of the printed structures' dimensional fidelity and feature resolution.

(5) Mechanical evaluation

Mechanical properties were assessed using an AG-20I universal testing machine (Shimadzu, Tokyo, Japan) at 200 mm/min on GB/T 1040.2-2006-compliant 1BA dumbbell specimens (Figure S8), with data averaged across quadruplicate measurements. Efficiency of welding, and physical reprocessing was quantitatively determined via tensile testing mentional aboved.

The dissipated energy (W_d, J/m^3) is calculated based on the cyclic tensile stress-strain curves under different strain levels, and its mathematical description is formulated as follows[6]:

$$W_d = \int_{\varepsilon_{min}}^{\varepsilon_{max}} \left(\sigma_{load}(\varepsilon) - \sigma_{unload}(\varepsilon)\right) d\varepsilon \quad (5)$$

where σ_{load} represents the stress along the loading path, σ_{unload} denotes the stress along the unloading path, and ε is the strain.

The hysteresis loss ratio (HLR) is determined from the cyclic tensile stress-strain curves under the same stress-strain condition, with its mathematical expression given by[6]:

$$HLR = \frac{W_d}{W_{total\text{-}in}} \times 100\% \quad (6)$$

$$W_{total\text{-}in} = \int_0^{\varepsilon_{max}} \sigma_{load} d\varepsilon \quad (7)$$

where W_d is the dissipated energy as defined above, and $W_{total\text{-}in}$ refers to the total input energy.

(6) PSA properties

Test of 180° peel strength for PSA performance was evaluated using HG-860 peel strength tester (Huaguo, Dongguan, China) according to the China Standard GB/T 2792-2014 at a fixed peeling rate of 100 mm/min. The interfacial adhesion toughness (Γ, J/m^2) was calculated as follows[7]:

$$\Gamma = \frac{2F}{\omega} \quad (8)$$

where F is the plateau force during peeling and w is the width of the specimen. Each adhesion result represents the average measurement of four samples having the same composition.

(7) Biodegradation testing

The biodegradability test was evaluated through soil burial tests. Initially, square specimens measuring 10×10×2 mm were printed using a 3D printer and placed in containers filled with garden soil. The specimens were buried 50 mm below the soil surface. Multiple sets of samples (including control samples) were prepared. Subsequently, the containers were placed in a humidity chamber with controlled temperature conditions set at 25°C and 30% relative

humidity, maintained for a duration of 45 days. At specific intervals, samples were retrieved, cleaned, and vacuum-dried at 25°C for 24 hours. The weights of the samples were measured before (W_{before}) and after (W_{after}) the biodegradation test, and the biodegradation rate (R_b, measured by the weight loss, %) was calculated using the following formula[8], while the reported data represent the average values obtained from four identical samples.

$$R_b=\frac{W_{before}-W_{after}}{W_{before}}\times 100\% \tag{9}$$

The microstructures of the samples before and after biodegradation were analyzed using a Leica MC170 HD metallurgical microscope (Leica Microsystems, Germany). Soluble residues from the leaching test were analyzed using an Ultimate 3000 UHPLC-Q Exactive liquid chromatography-mass spectrometry system (Thermo Fisher Scientific, Waltham, USA).

Table S1. The thermogravimetric datas of WHFA/HEA resins and pure HEA.

Sample	Initial decomposition temperature (℃)	The temperature at which the decomposition rate is maximum (℃)	Peak decompositi on rate (%/min)	The final carbon residue (%)
A1	314.87	395.52	0.65	0.99
A2	331.23	406.63	0.70	4.38
A3	358.32	413.84	0.76	5.82
A4	367.49	401.91	0.69	1.86
Pure HEA	370.03	418.56	0.89	2.58

Table S2. The glass transition temperatures (T_g) of WHFA/HEA resins and pure HEA.

Sample	Glass transition temperature (T_g, ℃)
A1	−24.5
A2	−18.2
A3	−8.8
A4	−5.1
Pure HEA	−4.9

Table S3. Tensile data of WHFA/HEA resins with varying WHFA content.

Sample	Tensile strength (MPa)	Breaking elongation (%)
A1	0.5412±0.0140	1092.58±10.96
A2	0.9618±0.0158	1184.66±16.58
A3	0.7944±0.0143	579.03±18.36
A4	0.7550±0.0161	539.19±15.66
E-WCO/HEA	0.5691±0.0159	595.35±18.59
SA/HEA	0.2234±0.0157	69.71±15.01
HEA	0.4157±0.0101	256.59±14.84

Table S4. Tensile data of of A2 elastomer in both pre-welding and post-welding states.

Sample	Tensile strength (MPa)	Breaking elongation (%)
pre-welding	0.9618±0.0158	1184.66±16.58
post-welding	0.2043±0.0244	523.99±22.76

Table S5. Tensile data of A2 elastomer across two reprocessing cycles.

Sample	Tensile strength (MPa)	Breaking elongation (%)
Original	0.9618±0.0158	1184.66±16.58
1st reprocessing	0.1646±0.0201	165.11±16.76
2nd reprocessing	0.1212±0.0201	90.07±19.76

Table S6. Interfacial adhesion toughness data of pristine HEA and A2 elastomer on diverse substrate materials.

Substrate materials	Interfacial toughness (J/m^2)	
	WHFA/HEA (A2 elastomer)	Pristine HEA
Steel	6.18±0.41	4.48±0.46
PMMA	8.00±0.40	5.08±0.41
Wood	15.46±0.42	4.58±0.40
Glass	32.60±0.38	11.10±0.40
PLA	14.48±0.40	3.86±0.41
Al	7.96±0.39	5.30±0.48
Pig skin	3.60±0.41	3.60±0.48

Table S7. Biodegradation data of A2 elastomer and pristine HEA during soil burial.

Time (days)	Weight Loss (%)	
	WHFA/HEA (A2 elastomer)	Pristine HEA
0	0	0
5	5.94±0.54	1.87±0.44
10	7.32±0.46	2.27±0.42
15	9.76±0.6	2.94±0.58
20	11.04±0.42	3.56±0.50
25	12.87±0.52	4.11±0.46
30	14.35±0.55	5.77±0.38

Table S8. Fatty acid compositions (wt.%) of WCO. Three main types of fatty acids: saturated (C n:0), monounsaturated (C n:1), and polyunsaturated with two or three double bonds (C n:2/3).

Fatty acid		Fatty acid compositions (wt.%)
Caprylic	C 8:0	0.3
Myristic	C 14:0	1.0
Palmitic	C 16:0	11.1
Palmitoleic	C 16:1	1.9
Stearic	C 18:0	2.4
Oleic	C 18:1	46.1
Linoleic	C 18:2	30.5
Linolenic	C 18:3	2.9
Arachidic	C 20:0	2.1

Table S9. The Lovibond color codes of WCO, E-WCO, WHFA and Liquid WHFA/HEA resin.

Sample	Y (Yellow)	R (Red)	B (Blue)	Light field	Dark field
WCO	2.2	3	3.1	0	0
E-WCO	7	1.2	0	0	0
WHFA	4.1	0.1	0.3	0	0
Liquid WHFA/HEA resin	13	5	0.2	0	0

Table S10. The viscosities of liquid WHFA/HEA resins and other control samples at room temperature.

Sample	Viscosity (mPa·s)
A1	42
A2	37
A3	32
A4	27
E-WCO/HEA	33
Pure HEA	20

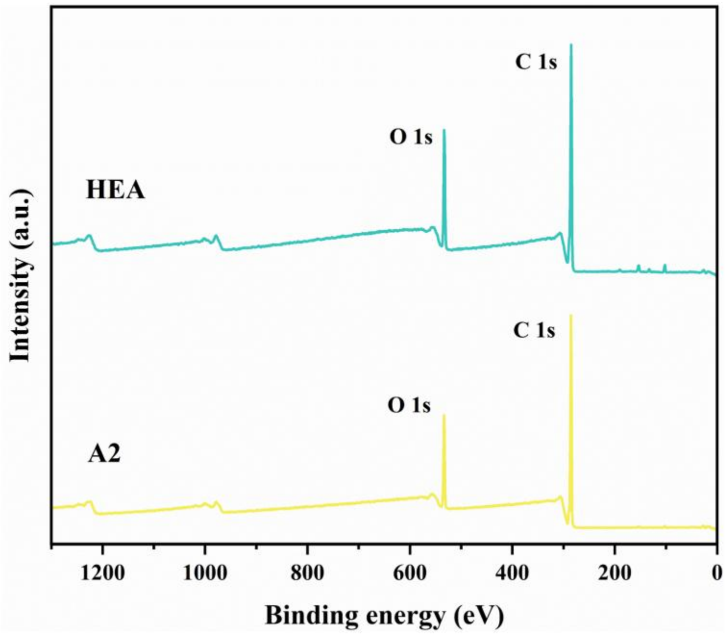

Figure S1. Full XPS spectra WHFA/HEA resin (A2 sample) and pure HEA.

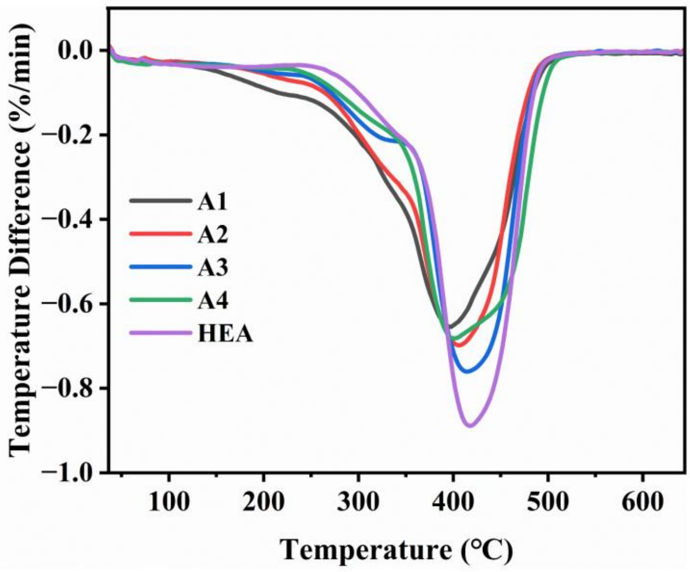

Figure S2. DTG curves of WHFA-HEA elastomers and pure HEA.

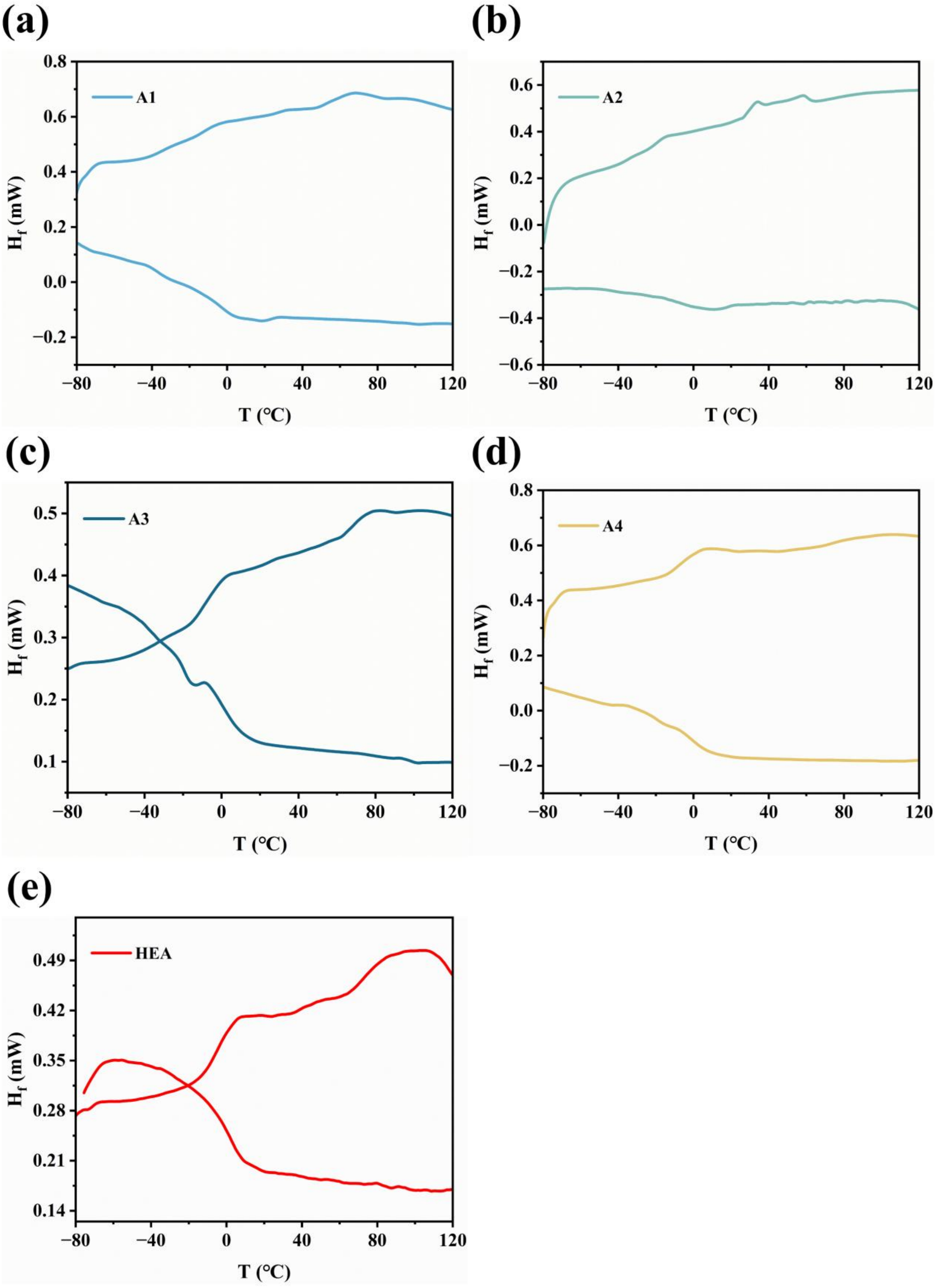

Figure S3. DSC curves of WHFA/HEA elastomer and pure HEA: (a) A1 sample, (b) A2 sample, (c) A3 sample, (d) A4 sample and (e) pure HEA.

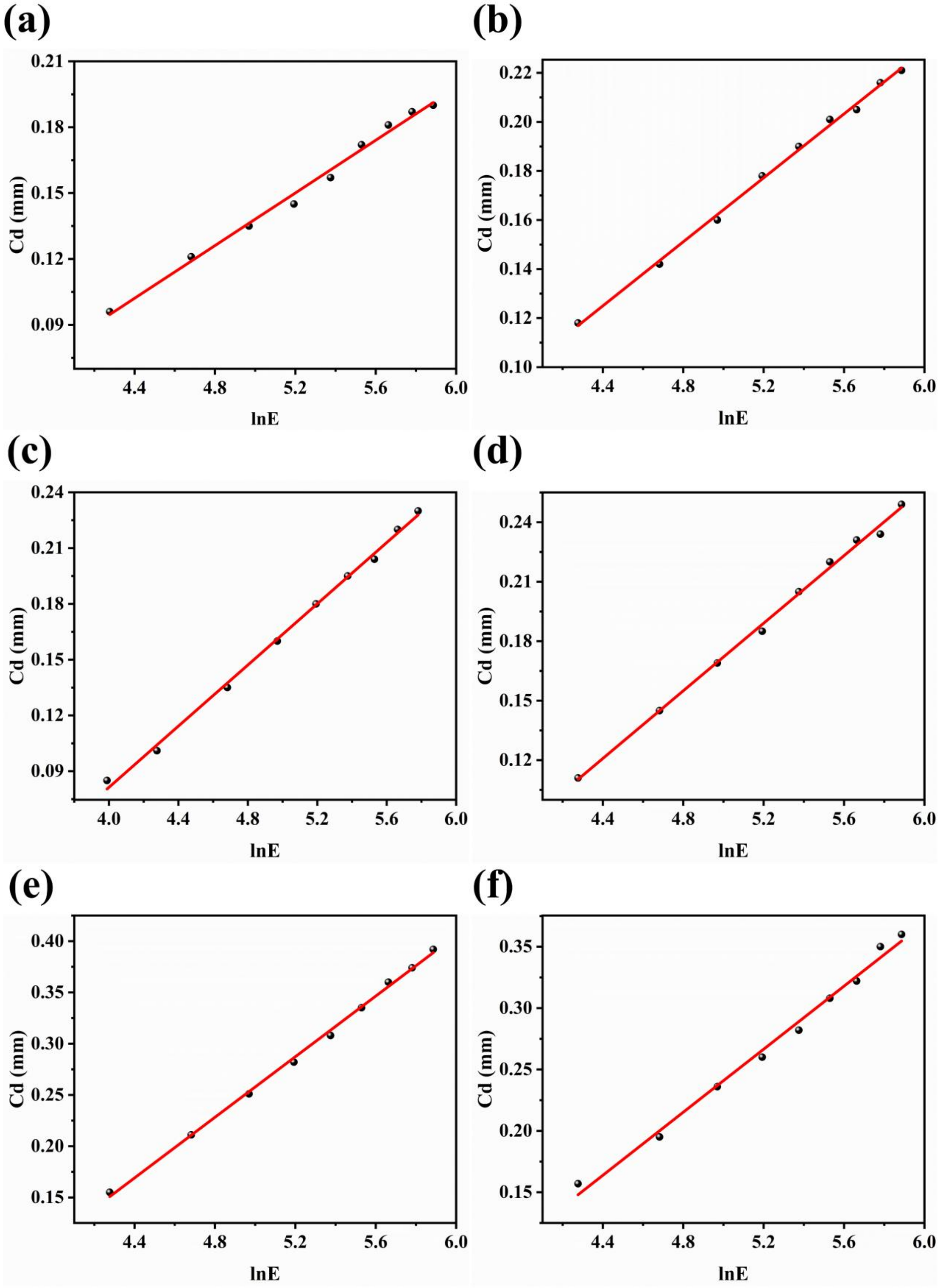

Figure S4. lnE-C_d plots of WHFA/HEA elastomer and other control samples: (a) A1 sample, (b) A2 sample, (c) A3 sample, (d) A4 sample, (e) E-WCO/HEA resin and (f) pure HEA.

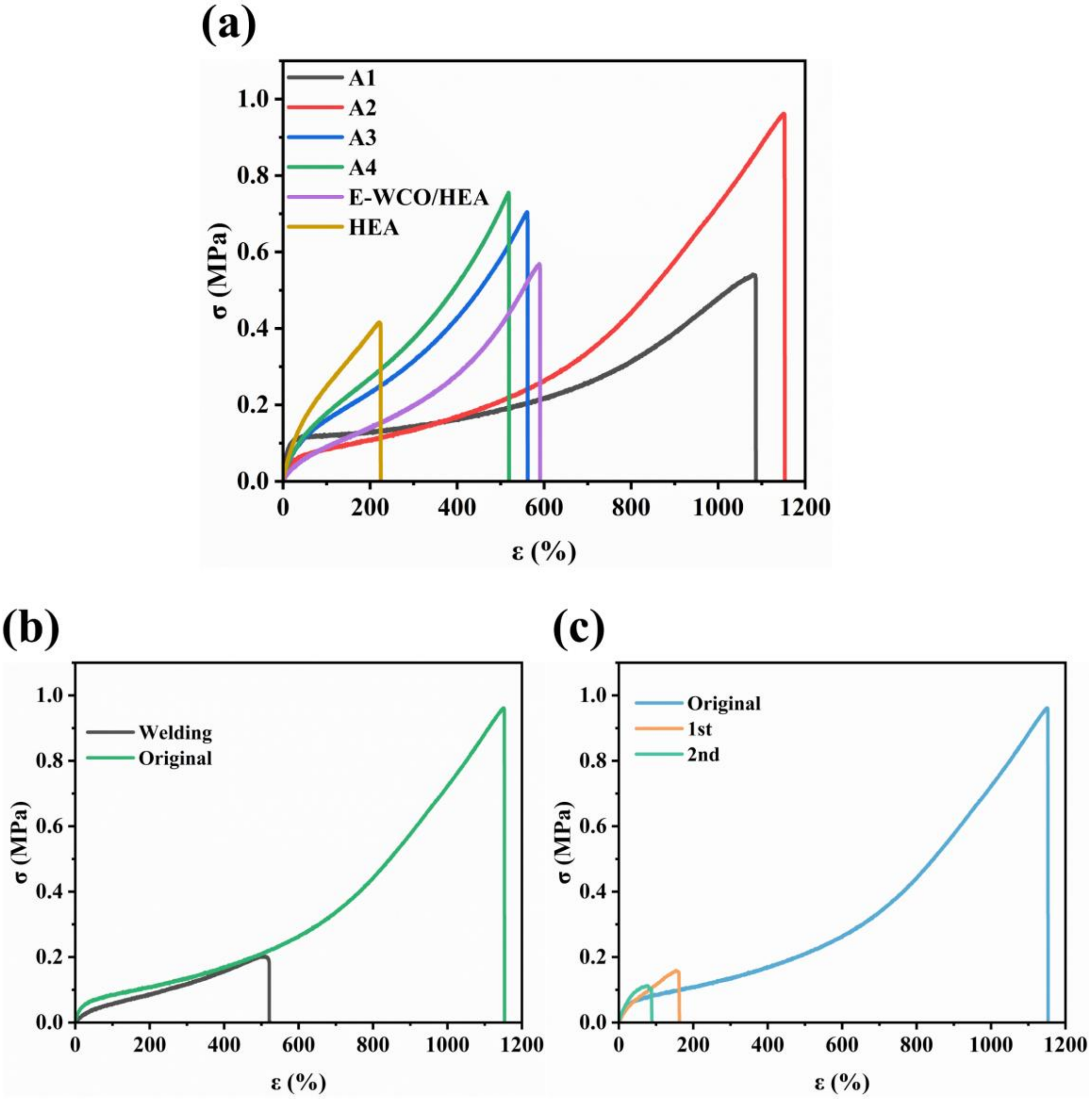

Figure S5. Stress-strain curves: (a) WHFA/HEA elastomers with different HEA contents (A1-A4 samples); pure HEA and E-WCO/HEA were used as control samples; (b) A2 elastomer before and after 12 hours of welding; (c) A2 elastomer before and after different physical reprocessing cycles.

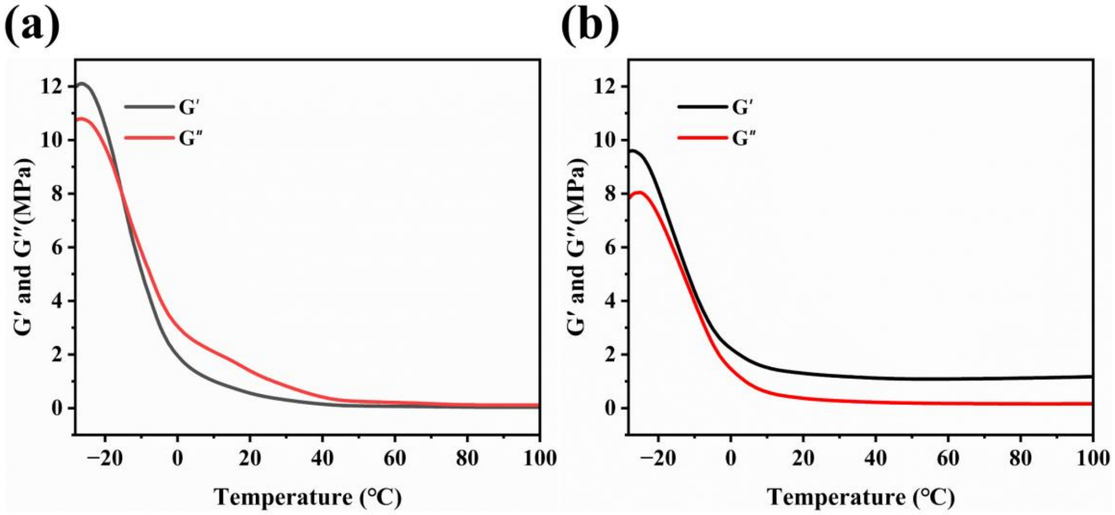

Figure S6. DMA curves of (a) WHFA/HEA elastomer (A2 sample) and pure HEA (b) .

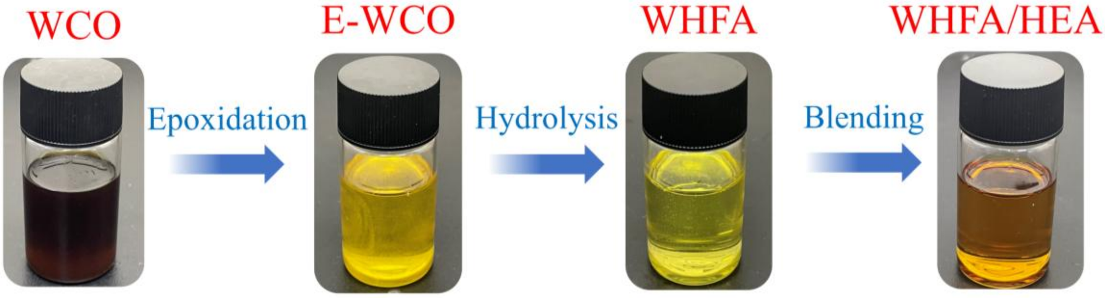

Figure S7. Photograph of WCO, E-WCO, WHFA (liquid form, melted at 60 ℃) and liquid WHFA/HEA resin showing the difference in their colors.

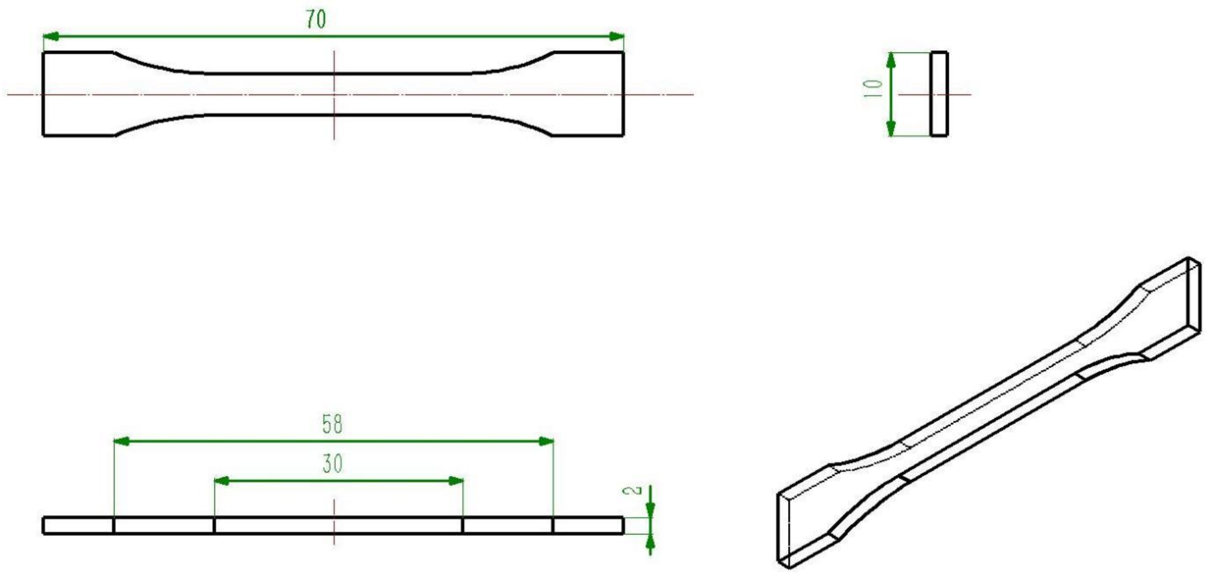

Figure S8. CAD model diagram of 1BA dumbbell-shaped spline according to the China Standard GB/T 1040.2-2006.

References

1. Liu, Y.; Liu, M. Y.; Fan, X. G.; Wang, L.; Liang, J. Y.; Jin, X. Y.; Che, R. J.; Ying, W. Y.; Chen, S. P. Four-Dimensional Printing of Multifunctional Photocurable Resin Based on Waste Cooking Oil. *Acs Sustainable Chemistry & Engineering* **2022,** 10, (49), 16344-16358.
2. Liu, Y.; Liu, M. Y.; Fan, X. G.; Wang, P. Y.; Chen, S. P. A 4D-Printable Photocurable Resin Derived from Waste Cooking Oil with Enhanced Tensile Strength. *Molecules* **2024,** 29, (9), 2162.
3. Wu, Y. P.; Li, R.; Wang, J. W.; Situ, Y.; Huang, H. A new carbazolyl-basedacylphosphine oxide photoinitiator with high performance and low migration. *Journal of Polymer Science* **2022,** 60, (1), 52-61.
4. Liu, M. Y.; Li, G. M.; Wang, P. Y.; Ying, W. Y.; Yang, Y.; Tang, C. Y.; Li, Y. Y.; Chen, S. P. Mechanically enhanced 3D printable photocurable resin composed of epoxy waste cooking oil and triethylene glycol dimethacrylate. *Journal of Polymer Research* **2024,** 31, (6), 177.
5. Bodor, M.; Lasagabáster-Latorre, A.; Arias-Ferreiro, G.; Dopico-García, M. S.; Abad, M. J. Improving the 3D Printability and Mechanical Performance of Biorenewable Soybean Oil-Based Photocurable Resins. *Polymers* **2024,** 16, (7), 977.
6. Fan, L. L.; Zeng, Z.; Zhu, R. X.; Liu, A. P.; Che, H. L.; Huo, M. Polymerization-Induced Self-Assembly Toward Micelle-Crosslinked Tough and Ultrastretchable Hydrogels. *Chemistry of Materials* **2022,** 34, (14), 6408-6419.
7. Wang, P. Y.; Sun, J. H.; Liu, M. Y.; Tang, C. Y.; Yang, Y.; Ding, G. Z.; Liu, Q.; Chen, S. P. Multifunctional 3D-Printable Photocurable Elastomer with Self-Healing Capability Derived from Waste Cooking Oil. *Molecules* **2025,** 30, (8), 1824.
8. Wu, B.; Sufi, A.; Biswas, R. G.; Hisatsune, A.; Moxley-Paquette, V.; Ning, P.; Soong, R.; Dicks, A. P.; Simpson, A. J. Direct Conversion of McDonald's Waste Cooking Oil into a Biodegradable High-Resolution 3D-Printing Resin. *Acs Sustainable Chemistry & Engineering* **2020,** 8, (2), 1171-1177.
